# Epitope detection in monocytes (EDIM) for liquid biopsy including identification of GD2 in childhood neuroblastoma—a pilot study

**DOI:** 10.1038/s41416-022-01855-x

**Published:** 2022-07-21

**Authors:** Matias J. Stagno, Andreas Schmidt, Jonas Bochem, Cristian Urla, Rupert Handgretinger, Karin M. Cabanillas Stanchi, Rafael Saup, Manon Queudeville, Jörg Fuchs, Steven W. Warmann, Evi Schmid

**Affiliations:** 1 Department of Pediatric Surgery & Pediatric Urology, Children’s Hospital, Eberhard-Karls-University Tuebingen, Tuebingen, Germany; 2 Department of Haematology and Oncology, Children’s Hospital, Eberhard-Karls-University Tuebingen, Tuebingen, Germany

**Keywords:** Tumour biomarkers, Paediatric cancer

## Abstract

**Background:**

Neuroblastoma (NB) is the most common paediatric extracranial solid malignancy. We analysed the role of the epitope detection in monocytes (EDIM) technique for liquid biopsy in NB patients.

**Methods:**

Tumour epitopes transketolase-like 1 (TKTL1), Apo10 (DNaseX) and GD2 were assessed: expression levels in seven NB tumour samples and five NB cell lines were analysed using RT-PCR and flow cytometry. LAN-1 cells were co-cultured with blood and assessed using EDIM. Peripheral blood macrophages of patients with neuroblastoma (*n* = 38) and healthy individuals (control group, *n* = 37) were labelled (CD14^+^/CD16^+^) and assessed for TKTL1, Apo10 and GD2 using the EDIM technology.

**Results:**

mRNA expression of TKTL1 and DNaseX/Apo10 was elevated in 6/7 NB samples. Spike experiments showed upregulation of TKTL1, Apo10 and GD2 in LAN-1 cells following co-culturing with blood. TKTL1 and Apo10 were present in macrophages of 36/38 patients, and GD2 in 15/19 patients. The 37 control samples were all negative. EDIM expression scores of the three epitopes allowed differentiation between NB patients and healthy individuals.

**Conclusions:**

The EDIM test might serve as a non-invasive tool for liquid biopsy in children suffering from NB. Future studies are necessary for assessing risk stratification, tumour biology, treatment monitoring, and early detection of tumour relapses.

## Introduction

Neuroblastoma (NB) is the most common extracranial solid tumour in infants and children and usually occurs within the first 2 years of life. The median age at diagnosis is 14 months [1]. It is classified as an embryonal neuroendocrine tumour, originating from neural crest progenitor cells and can be located anywhere along the sympathetic nervous system (e.g. the superior cervical ganglion, paraspinal sites, or the coeliac ganglia). The adrenal glands are most frequently affected (~50% of the cases) [2]. At the time of diagnosis, almost half of the NBs have metastasised, usually spreading to the bone marrow (ca. 80% of the cases), regional lymph nodes and bones, and also to the liver and skin [3, 4]. Neuroblastomas are generally classified as low-, medium- or high risk according to the criteria of the International Neuroblastoma Risk Group (INRG) [5]. Patients with high-risk neuroblastoma (HR-NB) require intensive multimodal therapy, however, the 5-year overall survival rate (OS) in HR-NB is below 50% [2, 6]. Diagnosis, staging, and risk stratification of NB is based on clinical signs, imaging, laboratory parameters, bone marrow aspiration, and histopathological and biological characteristics. MYCN amplification and loss of chromosome 1p are correlated with an unfavourable outcome [2].

Currently, non-invasive methods are being developed as diagnostic tools, treatment stratification or follow-up markers in solid tumours of children and for determination of treatment regimens in patients with NB [7, 8]. The epitope detection in monocytes (EDIM) test allows the detection of specific tumour markers for diagnosis or follow-up analysis of malignant and non-malignant tumours [9–11]. This method is based on macrophages (CD14+/CD16+), the number of which may be elevated in the blood of patients with diseases such as cancer, and which phagocytose parts of neoplastic cells and possibly extracellular vesicles (EVs) or circulating tumour cells (CTCs) [10, 12, 13]. Thus, tumour cell components accumulate in the activated macrophages and can then be extracted from the peripheral blood and analysed e.g. by flow cytometry via CD14 and CD16 [12, 13]. EDIM detection of TKTL1 and Apo10 has recently been presented as a sensitive and specific method for several tumours in adults [14, 15]. So far, there is no experience with EDIM blood test in paediatric solid tumours.

Transketolase-like 1 (TKTL1) is elevated in a variety of different tumour entities and has been correlated to tumour stage, aggressive behaviour, invasive growth, therapeutic resistance, and poor prognosis [9, 13, 16–29]. TKTL1 plays a central role in the promotion of aerobic glycolysis (Warburg effect). Through this, cell proliferation, angiogenesis (HIF1α), invasion and metastasis, repression of the immune system, therapy resistance and cell proliferation are supported [14, 26, 30]. Early detection and monitoring of malignancies by the EDIM-TKTL1 blood test has been analysed and evaluated for several solid tumour entities e.g. breast cancer or prostate cancer [14].

Apo10 is a specific epitope of the DNaseX (also: DNase-like 1). In normal cells, overexpression of DNaseX leads to increased internucleosomal DNA degradation and induction of cell death [31]. In malignant cells, this enzyme activity is inhibited and prevents the degradation of DNA despite elevated concentrations of DNaseX [14]. The EDIM-Apo10 analysis of DNaseX has been investigated and evaluated for several solid tumour entities [9, 13–15, 32, 33]. Its value as an identification marker for apoptosis resistance in pre-cancerous lesions or as a detection marker for invasive carcinomas has recently been shown [32].

The disialoganglioside GD2 is a glycolipid specifically expressed on cells of neuroectodermal origin such as neurons, skin melanocytes, and peripheral sensory nerve fibres; its expression on other cells is highly restricted [34]. It is uniformly expressed by neuroblastomas, most melanomas, and some other tumours [35–37]. Tumour-associated carbohydrate antigens such as GD2 have been shown to be involved in metastasation, tumour proliferation, and invasion. It plays an important role in signal transduction and cell adhesion [38]. High expression levels of GD2 in neuroblastomas (96%) and its restricted distribution in normal tissues make anti-GD2 antibodies potentially suitable as biomarker, which help to identify neuroblastoma early in its course and which enable therapies with maximal efficacy [39, 40].

Aim of this study was to assess the possible role of the EDIM technology in neuroblastoma and to establish a GD2-specific EDIM blood test in this tumour entity.

## Materials and methods

### Patient characteristics

Patients (0–21 years old) with histologically proven primary and/or recurrent NB (*n* = 38; Table 1) were prospectively recorded in this study. Children treated for non-oncological reasons (*n* = 37) served as a control group. Diagnosis of NB was histopathologically confirmed by the local Department of Pathology in Tuebingen as well as by the Reference Pathology of the GPOH (Pathology Kiel/Bonn). Patients with acute inflammation or immune-mediated diseases were excluded from this study. Written informed consent to participate in this study was obtained from all patients and healthy individuals or their legal representatives. The study was approved by the Ethics Committee of the Medical Faculty of the University Hospital Tuebingen (190/201B01 and 615/2015B02). A sample size of 34 will have 80% power to detect an effect size of 0.500 using a paired *t* test with a 0.050 two-sided significance level.

**Table 1:** Patient characteristics.

Characteristics	Number of patients *n* = 38	Number of healthy individuals *n* = 37	*P* value
	No. of patients (%)	No. of patients (%)	
Age (months)			
Mean	60 (range 24–168)	162 (range 60–204)	0.0001
Gender			
Male	24 (63.2%)	24 (64.9%)	0.1091
Female	14 (36.8%)	13 (35.1%)	0.1091
Stage			
I	1		
II	1		
III	10		
IV	22		
NMYC status			
Negative	33		
Positive	5		
Chromosome 1 (p36)			
Negative	9		
Positive	4		

*n* sample size, *No.* number.

### Cell lines and culture conditions

NB cell lines (LAN-1, SK-N-BE, IMR-32, LS, SH-SY5Y) were cultured in RPMI (Sigma Aldrich, Taufkirchen, Germany; Cat# R8758), supplemented with 1% penicillin/streptomycin (Sigma Aldrich, Cat# P4458) and 10% FBS (Biochrom, Berlin, Germany; Cat# S0115/0637T) in a humidified atmosphere containing 5% CO_2_ at 37 °C. All cell lines were regularly tested negative for *Mycoplasma spp*. (MycoAlert Mycoplasma detection kit, Lonza, Basel, Switzerland) and recently authenticated by STR.

### RNA isolation and real-time PCR

Determination of TKTL1 and Apo10 transcript levels was done using real-time PCR. Total cellular RNA was extracted from cell culture media using RNeasy Mini kit (Qiagen, Hilden, Germany; Cat# 74104) according to the manufacturer’s instructions. cDNA synthesis was performed using High capacity cDNA Reverse Transcription Kit (Applied Biosystems, Waltham, Massachusetts, USA; Cat# 4374966). PCR amplification of the genes TKTL1, Apo10 and TBP were set up in a total volume of 20 μl using 40 ng of cDNA, 500 nM (TKTL1, TBP) or 100 nM (Apo10) forward and reverse primer and 2× GoTaq® qPCR Master Mix (Promega Corporation, Madison, WI, USA; Cat# A6002) according to the manufacturer’s protocol. Cycling conditions were as follows: initial denaturation at 95 °C for 5 min (hot start), 95 °C for 15 s, followed by 40 cycles of 58 °C (Apo10) or 60 °C (TKTL1) for 30 seconds and 72 °C for 30 s. For the amplification the following primers were used (5‘−3′orientation):

TKTL1, forward: CGCCGAGCACTGCATAAA

TKTL1, reverse: CCACATAAGTGTTCCACCCAAA

DNaseX/Apo10, forward: AGCTGGTGTCTGTGAAGAGG

DNaseX/Apo10, reverse: CCGTGTAGACCTCAACCAAC

TBP, forward: GCCCGAAACGCCGAATAT,

TBP, reverse: CCGTGGTTCGTGGCTCTC.

Specificity of PCR product was confirmed by melting curve analysis. Real-time PCR amplifications were performed on a CFX96 Real-Time System (Bio-Rad). All experiments were done in duplicates. Amplification of the house-keeping gene *tbp* (TATA-binding protein) was performed to standardise the amount of sample RNA. Relative quantification of gene expression was achieved using the ΔCt method as described earlier [41].

### Flow cytometry—abundance of proteins in neuroblastoma cell lines

TKTL1, Apo10 and GD2 abundance were detected by flow cytometry. Cells were washed with cell wash buffer (BD Biosciences, Heidelberg, Germany) and were intracellularly stained with the TKTL1 (PE labelled; provided by Zyagnum), Apo10 (FITC labelled; provided by Zyagnum), and GD2 (APC labelled, provided by Prof. Handgretinger) antibodies at room temperature for 20 min. The samples were collected on a CantoII flow cytometer (BD Biosciences) and analysed using FACS Diva v8.0.1 cytometry analysis software (BD Biosciences).

### Flow cytometry—spike experiments

LAN-1 cells were seeded at a density of 1 × 10^4^ cells/100 µl into 96-well flat bottomed microtiter plates. After overnight adherence in a humidified incubator at 37 °C and 5% CO_2_, 100 µl of freshly drawn EDTA blood from healthy individuals were added either to the NB cells, to medium without cells or to empty wells. After co-culturing for 24 h in a humidified atmosphere containing 5% CO_2_ at 37 °C, cells were harvested and analysed via flow cytometry using the EDIM blood test technology. CD14/CD16-positive monocytes/macrophages were analysed for intracellular TKTL1, Apo10, and GD2 protein abundances.

### Flow cytometry—EDIM blood test

For the EDIM blood test, blood samples (2.7 ml) from neuroblastoma patients after chemotherapy or healthy individuals were collected in EDTA tubes (Sarstedt, Nümbrecht, Germany; Cat# 05.1167), anonymized, processed on the following day and blinded to the clinical data. After counting the cells via ADVIA 120 Hematology System (Siemens Healthcare GmbH, Erlangen, Germany), flow cytometry analyses of whole blood samples were performed as followed:

In total, 110 µl of blood was added to the surface marker mix in FACS tubes (BD Biosciences), mixed and incubated for 15 minutes at room temperature in the dark [CD14 (PerCP; #345786) and CD16 (–APC; #561304) for identifying macrophages [42, 43], and CD45 (APC-H7; #641417) as a selection marker for vital leukocytes, provided from BD Biosciences]. Then, 100 µl of IntraPrep reagent 1 (Beckman Coulter, Krefeld, Germany; Cat# A07803) was added, and the tubes were mixed manually and incubated again for 15 min in the dark. Following the incubation, samples were washed with 3.5 ml CellWASH (BD Biosciences, Cat# 349524), centrifuged at 300 × *g* for 5 min at room temperature and decanted. Thereafter, the tubes were incubated for 5 min and the pellets were carefully resuspended using a pipette. In the next step, 100 µl of IntraPrep reagent 2 (Beckman Coulter, Cat# A07803) was added to the tubes, the rack was shaken shortly, and then incubated for 5 min in the dark. The intracellular antibodies TKTL1 (PE), Apo10 (FITC), provided from Zyagnum AG, and GD2 (APC labelled) were added, and the tubes were incubated for further 20 minutes in the dark, washed twice with 3.5 ml CellWASH and then centrifuged at 300 × *g* for 5 min at room temperature. The pellet was resolved by “ratschen” and 300 µl BD CellWASH were added to the FACS tubes, incubated for 30 min, and the entire tube was measured (recorded until the sample was completely empty) using CantoII (BD Biosciences). Analysis was performed using FACS Diva software v8.0.1 (BD Biosciences). The commercial name of the EDIM blood test is “PanTum detect blood test” from the company Zyagnum AG with the same cut-off values for TKTL1, Apo10 and the combined score as previously described [15].

### Statistical analysis

Chi-square-tests (with Yates’ continuity correction) of GraphPad QuickCalcs were used for two-sample tests for equality of proportions and applied to the frequencies in patient characteristics and clinical parameters of the two study groups. All other data were tested for significance using unpaired Student’s *t* test or ANOVA analysis of variance (Bonferroni test and Dunnett test). To calculate the cut-off value for GD2 among NB patients and healthy individuals, a receiver-operating characteristics (ROC) analysis was performed and calculated with the Youden Index. Results with *P* < 0.05 were considered statistically significant. All figures show means of at least three individual experiments, with error bars representing SEM unless otherwise indicated. Graphs and statistical tests were created using GraphPad Prism 8 for Windows Version 7.03 (GraphPad Software Inc., La Jolla, CA, USA). Symbols indicate: *P < 0.05; **P < 0.01; ***P < 0.001; ****P < 0.0001.

## Results

### Patient characteristics

In this study, the patient group consisted of 38 NB patients with a median age of 60 months (range 24–168). In the control group, a total of 37 patients with a median age of 162 months (range 60–204) were enrolled. Statistical analyses of the equality of the proportions of the two study groups showed no significant differences in sex of the patients. However, a significant difference of the patient characteristics in age was detected (Table 1).

### Expression levels of TKTL1 and Apo10 in different neuroblastoma cell lines

**Fig. 1:**
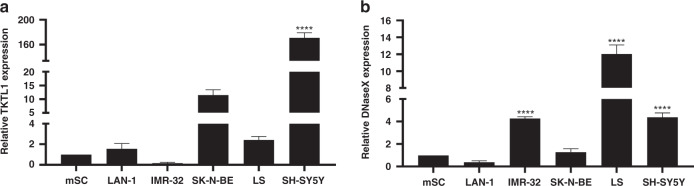
Expression of TKTL1 and DNaseX/Apo10 mRNA in neuroblastoma cell lines. Transcriptional expression of TKTL1 (**a**) and DNaseX/Apo10 (**b**) in various neuroblastoma cell lines relative to mesenchymal stem cells (mSC) as control (normalised to 1). TKTL1 and DNaseX/Apo10 mRNA levels were measured by quantitative real-time RT-PCR, with TBP as the internal control. The data are presented as SEM, *n* = 4, *P < 0.05, ****P < 0.0001 indicated statistical significance to mSC.

**Fig. 2:**
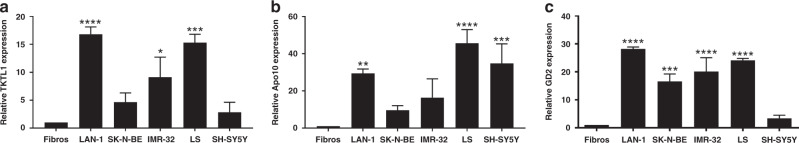
Protein expression of TKTL1, Apo10 and GD2 in various neuroblastoma cell lines. Relative TKTL1 (**a)**, Apo10 (**b**) and GD2 (**c**) protein expression in LAN-1 and SK-N-BE compared to fibroblasts as control (normalised to 1). Protein abundances were obtained using flow cytometry. Data are presented as SEM, *n* = 4, *P < 0.05, **P < 0.01, ***P < 0.001 and ****P < 0.0001 indicate statistical significance to fibroblasts.

In this work, we quantified TKTL1 and DNaseX/Apo10 at the mRNA level by RT-PCR in a panel of NB cell lines (LAN-1, IMR-32, SK-N-BE, LS, SH-SY5Y). Significantly increased expression levels of TKTL1 in the cell lines SK-N-BE and SH-SY5Y compared to mesenchymal stem cells (mSC) could be detected at the mRNA level (Fig. 1). However, DNAseX/Apo10 was significantly upregulated in LS and SH-SY5Y cell lines in comparison to mSCs. To foster our results, we also examined protein abundance in NB cells via flow cytometry. Protein abundance of TKTL1 in LAN-1, IMR-32 and LS cell lines were significantly upregulated (Fig. 2), as well as Apo10 in LAN-1, LS and SH-SY5Y cell lines. Significantly increased expression levels of GD2 in NB cell lines LAN-1, SK-N-BE, IMR-32 and LS cell lines compared to fibroblasts could also be detected.

### Co-culture experiments with whole blood and NB cell lines

**Fig. 3:**
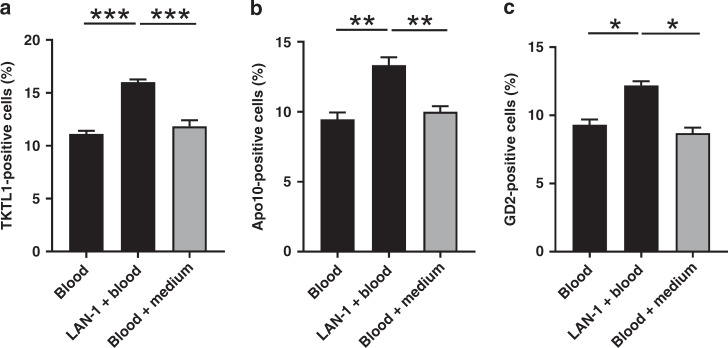
Tumour marker abundance in macrophages of co-cultured whole blood with neuroblastoma cell lines. The presence of TKTL1 (**a**), Apo10 (**b**) and GD2 (**c**) in MΦ was measured after using the EDIM test via flow cytometry. Co-culture experiments with LAN-1 show uptake of tumour markers in CD14+/CD16+ MΦ. Black and grey bars indicate internal control with (grey) and without (black) medium. Data are presented as SEM, *n* = 3; *P < 0.05, **P < 0.01, ***P < 0.001 indicates statistical significance.

To prove that macrophages phagocytose tumour cells and internalise them, we did several co-culture experiments. We selected the LAN-1 line for further co-culture experiments, which showed the highest levels of TKTL1 and Apo10 protein expression (Fig. 2). In order to exclude upregulation of TKTL1, Apo10 and GD2 in macrophages by the presence of medium or by incubation in the incubator, peripheral blood from the healthy individuals with DMEM Medium (with l-glutamine, P/S and FCS) were incubated for 24 h in the incubator at 37 °C and 5% CO_2_. We observed a significant upregulation of TKTL1, Apo10, and GD2 in peripheral blood (in macrophages) co-incubated with LAN-1 cells in comparison to peripheral blood cells alone or with peripheral blood cells incubated with DMEM Medium (Fig. 3). These findings confirm that the upregulation is not through incubation or medium alone.

### mRNA expression of TKTL1 and Apo10 in NB patient samples

**Fig. 4:**
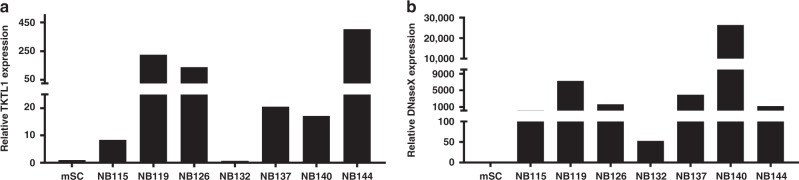
Expression of TKTL1 and DNaseX/Apo10 mRNA in patients with neuroblastoma. Transcriptional expression of TKTL1 (**a**) and Apo10 (**b**) in neuroblastoma samples. TKTL1 and Apo10 mRNA levels were measured by quantitative real-time RT-PCR, with TBP as the internal control.

Due to the sample size, not all patients were eligible for assessment of TKTL1 and DNaseX/Apo10 mRNA. Therefore we analysed seven neuroblastoma samples for TKTL1 and DNaseX/Apo10 mRNA. As illustrated in Fig. 4, there were higher mRNA levels of TKTL1 in 6/7 samples and Apo10 in 7/7 samples from patients with NB in comparison to control tissue (mSCs).

### EDIM blood test in NB patients

**Fig. 5:**
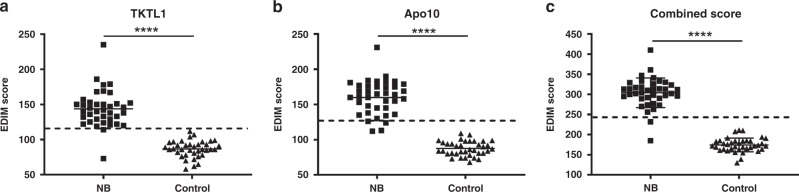
EDIM scores of patients and healthy individuals. TKTL1 EDIM scores (**a**), Apo10 EDIM scores (**b**), and the combined EDIM score (**c**) for the neuroblastoma (NB) and control group (control). For the EDIM scores, the respective marker abundances are measured in macrophages of the whole blood via flow cytometry. The dotted line indicates the cut-off value that is used to discriminate between neuroblastoma patients and control individuals. NB neuroblastoma patients (*n* = 38); control: control individuals (*n* = 37). ****P < 0.0001 indicates statistical significance to control.

Patients’ characteristics of the whole cohort are summarised in Table 1. In total, 36 of 38 NB patients (94.7%) showed positive (>119) EDIM-TKTL1 scores, and 34 of 38 patients (89.5%) showed positive (>129) EDIM-Apo10 scores. None of the control individuals was positive for both values. Median values of EDIM-TKTL1 and EDIM-Apo10 scores were significantly elevated in the NB group compared to healthy individuals (Fig. 5). Using the combined score (EDIM-TKTL1 and EDIM-Apo10; >248) 36/38 patients (94.7%) were positively detected. Median values of combined EDIM scores were significantly elevated in the NB group compared to the control group (Fig. 5). There were no correlations between TKTL1/Apo10 scores and patient characteristics (age, gender, stage) or tumour high-risk defining factors (MYCN, St. IV, Chr. 1; all *P* > 0.05).

### GD2 levels in EDIM blood test in NB

**Fig. 6:**
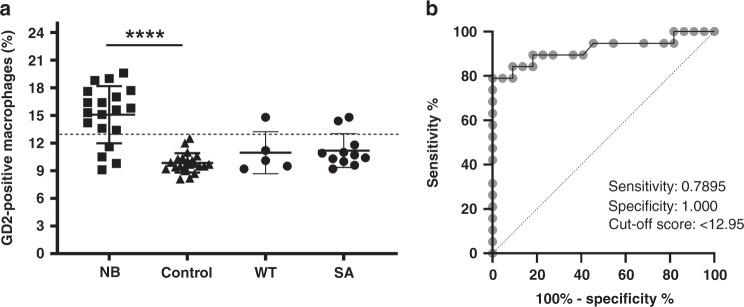
GD2 abundance in CD14^+^/CD16^+^ macrophages. **a** Comparison between GD2-positive macrophages of patients with neuroblastoma (NB), Wilms tumour (WT), sarcoma (SA), and control individuals (control). Whole blood from participants was analysed using the FACS-based EDIM test. The results are shown as a percentage of total macrophages. NB neuroblastoma patients (*n* = 19), WT Wilms tumour patients (*n* = 5), SA sarcoma patients (*n* = 11); Control: control group (*n* = 22). ****P < 0.0001 indicates statistical significance to control. **b** Receiver-operating characteristic (ROC) analysis of GD2 cut-off score in NB patients (*n* = 19) compared with healthy individuals (*n* = 22). The true positive rates (sensitivity) are plotted as a function of the false-positive rate (1—specificity) for measuring the cut-off point; ROC analysis for the diagnosis of NB shows the calculated cut-off value with the highest diagnostic accuracy of GD2 (cut-off score >12.95; sensitivity 0.7895, 95% CI 0.5667–0.9149; specificity 1.000, 95% CI 0.8513–1.000). The dotted line shows 95% CI.

After the initially examined patients had consistently shown a good expression of the proteins TKTL1 and Apo10, with which the EDIM technology was developed, we additionally carried out a respective analysis with regard to a GD2 expression in 19/38 patients. GD2 was chosen as a target because of its importance for the diagnosis and therapy of neuroblastoma and because it has never been described within the EDIM panel until now. Therefore, a separate evaluation of the data was performed. Together with these 19 patients, 22 healthy individuals were also evaluated as a control group for GD2 expression. For GD2 levels in the EDIM blood test, we included 19 NB patients (5 female and 14 male patients) with a median age of 48 months (range 24–156) as well as 22 control individuals (10 female and 12 male) with a median age of 164 months (range 24–240). Fifteen NB patients (79%) showed positive EDIM-GD2 values, whereas none of the healthy individuals (0%) showed a positive EDIM-GD2 level. Median value of the EDIM-GD2 was significantly elevated in the NB group compared to the control group (Fig. 6a). The receiver-operating characteristic (ROC) analysis (Fig. 6b) was performed, including all 19 NB patients compared with healthy individuals (*n* = 22). The analysis of the ROC curve showed that the best cut-off score for EDIM-GD2 is 12.95, resulting in a sensitivity of 78.95% (56.67% to 91.49%) and a specificity of 100% (85.13–100.0%). The area under the curve (AUC) was 0.9199 (*P* < 0.0001).

To demonstrate that GD2 is tumour-specific, we analysed additional patients with Wilms tumours (*n* = 5) and sarcoma (*n* = 11), for GD2. Among them, 4/5 Wilsmtumor patients (80%) and 9/11 sarcoma patients (81.82%) showed a negative value of GD2 with a cut-off score of 12.95.

## Discussion

The clinical behaviour and course of NB vary depending on clinical and histological/biological characteristics. A large proportion of NB detected in newborns (stage 4S) differentiates or regresses spontaneously without treatment. Other children may develop a broad spectrum of disease ranging from local tumours with varying extension to metastatic tumours with poor outcomes despite aggressive multimodal therapy. Although new therapies are being developed, there is still a need for an optimised recurrence monitoring method throughout therapy. Current methods include MRI and CT, which require the undesirable use of anaesthetics in younger children. Furthermore, local relapses are regularly difficult to detect early on imaging because of extensive postoperative changes of the tissue (especially scarring and inflammation) and they are detectable only from a certain size on. The EDIM blood test could therefore possibly provide a new, non-invasive diagnostic tool for treatment planning, risk stratification, and identification of follow-up markers in paediatric solid tumours. Recent studies have shown that TKTL1 plays an important role in the development and progression of human tumours, and has been detected and correlated with several types of cancer [44].

In the present pilot study, we evaluated the EDIM blood test in 38 patients with clinically proven NB and in 37 healthy control individuals. Our study shows that, 94.7% of patients with neuroblastoma had positive (>119) EDIM-TKTL1 scores, and 89.5% had positive (>129) EDIM-Apo10 scores. Using the combined score (>248) of EDIM-TKTL1 and EDIM-Apo10, 94.7% (36/38) of NB patients were positively detected. This is in agreement with previous findings, where elevated TKTL1 and Apo10 levels are correlated with the presence of prostate cancer and oral squamous cell carcinoma [11, 13]. Jansen et al. also showed that the combined TKTL1 and Apo10 EDIM blood test was positive for a male patient nine months before colon cancer metastasis was detected through MRI [9, 13]. Two NB patients in our series had negative EDIM scores before tumour resection. One patient, who also suffered from opsoclonus–myoclonus syndrome (OMS), had received a dexamethasone pulse some days before the blood was drawn. A dose-dependent immunomodulatory effect of dexamethasone on monocytes has been previously described [45]. However, it needs to be clarified in future investigations whether this medical treatment constantly interferes with the EDIM method in NB patients with OMS. The second NB patient, in which we observed a negative EDIM score, had a completely necrotic tumour after chemotherapy with <1% vital tumour cells suggesting the necessity for correlating EDIM results with pathological findings in cases of post-chemotherapy evaluations using this method. Interestingly, no false-positive EDIM values (TKTL1 and Apo10) were detected in our healthy control group. With a sensitivity of 94.74% (CI 82.71–99.06%) and specificity of 100% (90.59–100%), the combined EDIM blood test seems to be a reliable liquid biopsy technique, which may be used for the detection of NB and other paediatric solid tumours. Future studies should assess the comparability of MRT/CT and the EDIM blood test, which could optimise this non-invasive recurrence monitoring method.

In this study, we quantified the TKTL1 and DNaseX/Apo10 mRNA levels of seven neuroblastoma patients using RT-PCR. Due to a limited tumour material availability, not all patients were eligible for assessment of TKTL1 and DNaseX/Apo10 mRNA. All seven patient samples showed higher DNaseX/Apo10 and 6/7 showed higher TKTL1 mRNA levels compared to human mesenchymal stem cells (mSCs). The majority of NB cells showed increased expression of TKTL1 and DNaseX/Apo10.

In the last step of apoptosis in normal cells, chromosomal DNA is cut into 300 base-pair fragments by the enzyme DNaseX. Malignant cells produce inhibitors of DNaseX, which prevents the cells from entering apoptosis and causes the accumulation of DNaseX within the nucleus [13]. Previous studies additionally indicated that TKTL1 protein and Apo10 epitope expression levels are elevated in tumour cells [13, 15, 21] and that TKTL1 may play a role as a controlling enzyme for cell cycle, glucose metabolism, invasion, metastasis and therapy resistance in tumours [21, 46, 47]. As mentioned above, we observed overexpression of TKTL1 and DNaseX/Apo10 at the mRNA and protein level in cell lines, and even higher overexpression in NB tumour specimen. Therefore, further studies on a possible impact of TKTL1 on the cellular and clinical processes mentioned above should be performed.

Since activated macrophages (CD14^+^/CD16^+^) phagocytose entire tumour-derived protein epitopes, other tumour-associated proteins may be detected via EDIM technology using specific antibodies as well. GD2 is an attractive target for the EDIM blood test because its expression is mostly restricted to malignant cells such as NB, most melanomas, Ewing sarcoma, and osteosarcoma [35–37, 48, 49]. Since GD2 has never been investigated for liquid biopsy in the EDIM blood test as a biomarker for NB, we analysed 19 NB patients and 22 healthy control individuals. We observed for the first time that 79% (15/19) of NB patients showed elevated GD2 levels. In contrast, 1/5 Wilms tumour patients (20%), 2/11 sarcoma patients (18.2%) and none of the patients of the control group showed a false-positive value for GD2. This is in agreement with previous immunohistochemical studies, which showed that GD2 has a high level of expression in NB patients [40, 50]. Due to the stressfulness of invasive biopsies and the fact that not many paediatric solid tumour biomarkers are known, the use of GD2 in liquid biopsy would improve the monitoring of NB. Based on the discovery that tumour-specific tumour markers can be detected with this method, the EDIM blood test might contribute to establishing a tumour-specific diagnosis in combination with imaging studies and might serve as a biomarker for follow-up examinations (to detect response to therapy and tumour relapse) in other paediatric solid tumours. In contrast to serum biomarkers, which are diluted by the blood volume, the biomarkers in the EDIM blood test are concentrated in the monocyte/macrophages. The utilisation of monocyte/macrophage cellular debris ingestion can have a significant impact on the specificity and sensitivity of such a test. In accordance with other reports of EDIM blood test measurements in cancer patients, it seems that the EDIM blood test with the NB marker GD2 might serve as a sensitive and non-invasive diagnostic tool to identify the presence of tumours and differentiate the type of tumour that is present. It is thus worthwhile to evaluate the EDIM blood test with GD2 in a clinical setting. Future studies should examine the difference between the EDIM blood test using the biomarkers TKTL1, Apo10, and GD2 and typical immunohistochemistry results. Finally, detection of GD2 using this technology might contribute to early identifying NB patients as being susceptible to anti-GD2 immunotherapy.

The EDIM technology is based on the invasion of monocytes and macrophages in solid tissue. During invasion, monocytes and macrophages phagocytose cellular debris (such as tumour debris), digest the cellular components and then return to the bloodstream [10, 12, 51]. Activated macrophages that circulate in the blood and harbour tumour-derived protein epitopes (possibly also from CTSs or EV’s) can be detected by antibodies (CD14 and CD16) via the non-invasive EDIM technology. To investigate whether elevated TKTL1, Apo10 and GD2 levels in the EDIM blood test are caused by biomarker internalisation by phagocytosis or by an upregulation that triggered in response to a tumour, we performed co-culture experiments. All biomarker levels (TKTL1, Apo10 and GD2) were significantly enhanced after incubation with tumour cells (LAN-1). When only incubating with medium there was no significant upregulation of the 3 biomarkers (compared to blood alone). These data show for the first time that macrophages phagocytose NB tumour cells and internalise them, and that TKTL1, Apo10 and GD2 are detectable in the EDIM blood test.

In conclusion, the EDIM liquid biopsy technology was able to differentiate between neuroblastoma patients and healthy individuals with a high sensitivity and specifity. Reproducible results were obtained from neuroblastoma tissue samples and cell lines fostering our clinical findings. This method might serve as a tool for treatment monitoring and early relapse detection for this tumour entity. However, further studies should be performed in a larger patient cohort as well as at different time points to confirm these results and to address unanswered questions, such as the association with tumour histology.

## Supplementary information

Reporting Summary

## Data Availability

The datasets used and/or analysed during this study are available from the corresponding author on reasonable request.
